# Implementing AI in Radiotherapy: Insights From the Healthcare Professions

**DOI:** 10.1111/1467-9566.70190

**Published:** 2026-04-22

**Authors:** Juan I. Baeza, Jon Hindmarsh, Angie A. Kehagia, Anna Barnes

**Affiliations:** ^1^ King's Business School King's College London London UK; ^2^ King's Technology Evaluation Centre (KiTEC) King's College London London UK

**Keywords:** artificial intelligence (AI), digital health, healthcare professionals, professions, radiotherapy

## Abstract

The potential for artificial intelligence (AI) to transform healthcare has been of growing academic, policy and professional interest. Various studies have reported on the perceptions of the potential for AI in healthcare. However, fewer studies have examined the lived experiences that healthcare professionals (HCPs) have with the use of AI tools. One area where AI has been implemented is in the auto‐contouring of organs‐at‐risk (OAR) for cancer treatment. In this study, we interviewed 32 HCPs involved in cancer treatment using these AI tools, across five different regional cancer centres in England. In contrast to studies that explore professionals' perceptions of the future possibilities of AI—which often focus on fear and concern—our respondents report very positive experiences. We find that the AI tools offer opportunities to enhance their professional autonomy by re‐focusing, on what they consider to be more expert activities. Our findings reveal the enduring value of insights from the sociology of the professions in the age of AI, and evidence the importance of a *partial* discard of tasks. They also show the relevance for emerging sociologies of AI to appreciate both the particularities of the AI tools in use, and the professions and practices at work.

## 1 Introduction

The potential for artificial intelligence (AI) to revolutionise healthcare has been of increasing interest over recent years (for notable reviews, see Siala and Wang 2022; Kusta et al. 2024; Vo et al. 2023). Indeed, it is argued that, ‘we are on the precipice of an artificial intelligence (AI) revolution that could transform care for patients’ (Darzi 2024, 105). However, it is known that the practical implementation of some AI tools can be problematic when vendors are ‘confronted with varied implementation contexts that render the spread of technology across organisations highly complex’ (Gillner 2024, 2), AI implementation is seen as ‘more complex than other IT innovations’ (Neumann et al. 2024, 134), and disruptions are seemingly inevitable (Carboni et al. 2023). In addition, there is a significant body of work that has revealed how professionals often resist, reorientate or workaround technological changes, including the introduction of AI tools, perceiving new tools as devaluing existing skills and threatening professional jurisdictions (Pachidi et al. 2021; Scarbrough et al. 2024).

Given the inchoate status of many AI systems in healthcare, the majority of social scientific studies focus on *perceptions* of healthcare professionals (HCPs) (e.g., Vo et al. 2023; Lombi and Rossero 2024) or wider discourses on the *possible futures* for AI (e.g., Watson et al. 2025). Therefore, as (Shinners et al. 2020, 1225, emphasis added) suggest, ‘more research is needed to examine *the experiences* and perceptions of healthcare professionals using AI in the delivery of healthcare.’ We sought to answer the focused research question: ‘What are the lived experiences of healthcare professionals of AI tools for contouring organs‐at‐risk (OAR)?’

Interestingly, and in contrast to many studies of AI deployment in healthcare, professionals were overwhelmingly positive about the introduction of this tool, a sentiment that cut across three broad professional categories located in five different medical centres.

The paper interrogates and explains this finding by drawing on key insights from the sociology of the professions, and the differing perspectives on the potential challenges new technology such as AI tools present to HCPs. For example, Abbott (1988) and Freidson (1970) argue that professionals claim jurisdiction over specific social problems by asserting their expertise as most appropriate. They highlight the importance of authority—the right to define and solve problems—and autonomy—the freedom from external interference—as central to professional identity. (Abbott’s 1988, 316) concept of jurisdiction is a useful way of examining the introduction of this new technology as, ‘it shows how professions both create their work and are created by it’. The new technology explored in this paper could have been treated by the professionals as claiming expertise in, and responsibility for, one of their core professional tasks. As such, the AI technology is an example of what Abbott calls an internal source of disturbance, which can lead to disputes over jurisdictional boundaries by challenging the profession's control over the technical and formal content of their work. However, in our case, this does not happen and this paper explores reasons for this.

The paper presents an empirical investigation of professional work and AI implementation that rests on in‐depth, qualitative interviews with professionals involved in organs‐at‐risk (OAR) contouring. The findings offer two key contributions. First, they develop our understanding of the professional dynamics involved in the deployment of new AI technologies in healthcare, by drawing on key insights from the sociology of the professions. Second, they present a broader challenge to sociological studies of technology to recognise that AI is not one homogeneous technology, and so, to fully appreciate the ‘domestication’ (Johannessen et al. 2024) of these tools, we need to interrogate their particularities in combination with a richer examination of the practices and professions involved at the site of deployment. Together, our findings illustrate the importance of examining professional, organisational and clinical contexts when studying the impact of different AI tools into healthcare settings.

## 2 Professional Dominance, Jurisdictions and New Technologies

In the social scientific literature on health and care, the work of Abbott (1988) and Freidson (1970) have been highly influential in understanding how and why certain occupations achieved professional dominance. Freidson argues that medicine achieved a unique position of dominance in modern societies by controlling the terms of its work and resisting external interference. Crucially, he distinguishes professions from mere technical expertise, emphasising that autonomy is socially constructed and maintained through institutional arrangements. This is similar to Pakarinen and Huising's (2025) distinction between substantialist and relational views of expertise. They reject the substantialist perspectives (e.g., Frey and Osborne 2017; Susskind and Susskind 2015) that view expertise as a simply bounded and codifiable substance that is separable from its social context. Instead they argue for a relational perspective, where expertise is constituted through social interactions and contextual interdependencies.

Abbott provides the conceptual apparatus to examine the principal means through which professions sustain dominance, namely ‘professional autonomy (which) raises questions about the degree to which professions control their work and self‐govern’ and ‘professional authority (which) raises questions about whether professions still dominate the work of healing our bodies’ (Huising 2023, 456). When work is treated as an ecology (Huising 2023), professions can be seen to have jurisdictions over a dynamic array of tasks, where the boundaries to those jurisdictions are managed in and through negotiations between different professions (Huising and Pakarinen 2025). Abbott (1988) highlights how the dominance of professions is sustained through ‘boundary work’ to manage relationships between groups in the field of action. Many studies have unpacked the boundary work undertaken by HCPs in maintaining, expanding, adjusting or securing their domains of jurisdiction in light of new developments (Abbott 1988; Allen 1997; Currie et al. 2012; Håland 2012; Kessler et al. 2015; Langley et al. 2019).

Professions work to protect and expand their jurisdictions over core and complex tasks, and it is argued that ‘an occupational group's survival depends upon its ability to maintain jurisdictional control over its task domain’ (Kahl et al. 2016, 1103). However, (Abbott 1988, 72) also stresses that it is important for professionals to pass on ‘dangerously routine work’ to assistants to maintain the expert nature of their work. This resonates with earlier work by Hughes (1951) and Bosk (1979) who argued that professions maintain status and legitimacy by outsourcing undesirable aspects of their work. Hughes differentiated between professional work versus menial and unpleasant labour that is delegated to less powerful subordinates, such as nurses or healthcare assistants. Similarly, Bosk demonstrated that, in surgical hierarchies, senior surgeons claim discretionary judgement and complex decision‐making as markers of expertise, clearly distancing themselves from other routine tasks that are framed as subsidiary responsibilities that can be delegated to more junior staff. (Kirkpatrick et al. 2011, 492) nicely summarise this claim, stating that ‘professions seek to advance their aims through the acquisition of high status roles and skills, while at the same time delegating less desirable tasks to subordinate groups.’

In an empirical study that builds on Abbott's insights, Kessler et al. (2015) explored the impact of the new healthcare assistant role on the nursing profession in England, in which they considered the import of a professional logic of specialist expertise. This logic, they argue, encourages ‘the profession to discard routine tasks’ (737) while at the same time sharpening ‘the exclusive right to perform a tightly defined set of advanced technical tasks’ (739).

In relation to technological change, four decades ago both Cockburn (1985) and Barley (1986) emphasised the critical importance of professionals and the ways in which they accommodate or resist technology projects. For example, Cockburn (1985) charted the introduction of Computed Tomography (CT) scans and linear accelerators into radiography, noting how ‘technological change causes tensions and movement in the relationships between these various groups of professional and technical employees’ (Cockburn 1985, 116). Although technologies have changed in the past 40 years, boundary work and interprofessional contests persist.

In Noordegraaf's (2020) contributions to the sociology of the professions, he highlights the loss of protective shields covering the professions due to pressures of economic, socio‐cultural and, indeed, *technological* change. He argues that professionalism is not immune to these outside pressures, but within this context, he asks ‘the question of how professional action can be related to others and outsiders and remain ‘knowledgeable’, ‘autonomous’ and ‘authoritative’ at the same time’ (Noordegraaf 2020, 205). He suggests that rather than being detached from those outside pressures, there is a need for studies to examine the ways in which professions retain their position through new or revised forms of connection to clients, stakeholders, and other influences.

A recent study by Scarbrough et al. (2024) examined some of these dyanmics, when exploring the views of radiologists, service managers and healthcare leaders on the introduction of AI into diagnostic radiology. In their sample of radiologists, AI was perceived as an, ‘antagonistic agent that interfered with their work roles and relationship with patients, constrained their agency, and challenged their professional jurisdiction’ (Scarbrough et al. 2024, 15). AI was treated as an ‘epistemic technology’ offering claims to expertise that are seen to question or challenge professional authority, and thereby threaten jurisdictional boundaries.

Furthermore, the ‘opacity’ common to many AI technologies has been shown to generate uncertainty in professionals because the AI results or recommendations can diverge from professional judgement without offering (adequate) reasoning (Lebovitz et al. 2022; Hartswood et al. 2003). For example, Lebovitz et al.’s (2021) study into the implementation of several AI tools into a US hospital reported that the machine's diagnoses often conflicted with that of the radiologists, as the AI tools' *know‐what* conflicted with the professionals' *know‐how*, which was not only based on the images but drew on their other knowledge of the patients, such as their medical history and other test results that helped them set the images in a much wider and richer context than the AI. These stories of HCP's fear and resistance to AI are common (e.g., Carboni et al. 2023; Kusta et al. 2024).

Scarbrough et al. (2024) also report that, in contrast to the views of the front line professionals, healthcare leaders welcome the potential transformational power of AI tools and healthcare managers hope to deliver service efficiencies in the form of increased and/or quicker patient throughput. This presents a contest between the *potential* service efficiencies and savings that might be gained by adopting these AI enabled technologies and the resistance to their implementation by HCPs who consider them a threat to their status and jurisdiction.

Organs‐at‐risk (OAR) contouring is a core task within the jurisdictions of the key professions involved in the delivery of radiotherapy. The AI tools we investigate make a claim on this task which could result in a jurisdictional challenge to the work of those HCPs. It is this jurisdictional tension that we explore in this paper. An understanding of professional authority and autonomy provides a valuable lens to interrogate why and how the HCPs in our study embraced the introduction of this new AI technology, in spite of its seeming threat to their expertise. This demands consideration of the practices and interests of multiple professions, as well as the tools used in the performance of the task (cf. Barrett et al. 2012; Eyal 2013).

## 3 AI and Auto‐Contouring ‘Organs‐at‐Risk’

The empirical focus for our study concerns radiation therapy for cancer treatment. Patients arrive at the radiotherapy treatment unit with a cancer diagnosis. The patient's journey begins with a radiotherapy planning appointment during which they will have a CT scan. Over a period of 2 weeks, that scan is used to build an individualised radiotherapy plan. Based on the treatment plan, both the dose and shape of the beam are adjusted so as to optimally concentrate radiation delivery to the cancer locus and spare adjacent healthy structures, known as ‘organs‐at‐risk’ (OAR). Protecting OAR from radiation exposure has vital functional implications and can be a significant determinant of quality of life post‐radiotherapy. This is achieved through contouring, or ‘drawing,’ the boundaries of OAR on each of up to a hundred 2D renderings that make up a 3D CT image. It is important to note that while OAR contouring is performed by a range of HCPs in the service—including specialised radiographers, dosimetrists and clinical scientists—it is the oncologist who retains the responsibility for evaluating and approving all contours and the treatment plan as the patient's responsible clinician.

AI tools, using deep learning methods, have recently been implemented for OAR auto‐contouring (Meyer et al. 2018) which draw the contours around different OAR structures. There has been some research into the implementation of these technologies, but it is quite limited (Strohm et al. 2020). Some studies have reported that the tools yield good quality contours and reduce inter‐ and intra‐observer variability (Doolan et al. 2023), but the key focus has been on the time saved in doing contouring work (Montague et al. 2024; Kiljunen et al. 2020; Zabel et al. 2020; Oktay et al. 2020).

HCPs involved in contouring work have been generally favourable of the introduction of these technologies. One survey found that 78% of members of the UK Faculty of Clinical Oncology report a positive impact on their practice, citing reductions in contouring time as a reason (Hindocha et al. 2023), which is in keeping with views expressed by medical physicists, therapeutic radiographers and radiation oncologists in other countries (Brouwer et al. 2020; Rainey et al. 2022; Mugabe 2021). However, some surveys note that the majority of clinicians report the need to amend AI contouring outputs, questioning the extent of the time savings (Montague et al. 2024). Others warn of negative impacts if the time saved by these new technologies simply leads to higher workloads through increases in case volume (Hindocha et al. 2023). These survey‐based studies suggest the need for a more in‐depth and qualitative understanding of how professionals reason about their experiences with these AI tools.

## 4 Methods and Approach

This study is part of a wider evaluation of AI tools in eight NHS Foundation Trusts across England. All participants worked in the UK National Health Service (NHS) and provided informed consent. Participants were informed that confidentiality and anonymity would be maintained and no patient data were collected. The study received university ethical approval and was also approved by each of the interview sites. The five sites in this qualitative study are leading regional cancer treatment centres in different parts of England and were chosen as they include a range of different AI tools that have been deployed in the NHS. As regional cancer treatment centres, they all have similar modes of operation and had at least 12 months' experience in using AI tools in OAR contouring. Most of the professionals interviewed had experienced the transition to the use of these tools, although a small number of more junior staff had only ever experienced contouring work with AI tools.

The framework for the purposive sample was agreed by all authors to ensure that a range of roles and levels of seniority would be represented at every site. Two authors (anon and anon) contacted respondents to invite them for interview by email. They were either interviewed (by anon or anon) in person in their workplace or remotely via MS Teams. The interviews lasted between 30 and 90 min, were audio recorded, transcribed in full and then analysed using NVivo. All the respondents interviewed regularly undertake, or oversee, contouring work. In total, 32 interviews were carried out, with the professional roles in Table 1 below broadly defined as clinical scientist, radiographer/dosimetrist or oncologist, to preserve participants' anonymity.

**TABLE 1:** Interview sample.

Professional role	Site A	Site B	Site C	Site D	Site E	Total
Clinical scientist	4	1	1	2	1	9
Radiographer/dosimetrist	1	4	4	3	3	15
Oncologist	0	0	3	2	3	8
Total	5	5	8	7	7	32

The purposive sample across the five geographical sites allowed the research team to explore how the three key professional groups reported on the implementation and experiences of using AI tools and to ask the relevant ‘how’ and ‘why’ questions that emerged (Strauss and Corbin 1998).

We adopted a practice‐oriented research perspective (Nicolini 2013), in that we were concerned with professionals' ongoing work practices, and how these shaped their situated use of, and reasoning about, new technologies. Therefore, our interviews with professionals focused on the work of contouring; the role that the AI tool plays in this contouring work; the position of contouring work in relation to other activities in their working days; and the changes they experienced in relation to work practices over periods of time (e.g., before and after the introduction of AI). Respondents were also asked about the implementation process of the AI tool and their perceptions of AI tools in general.

The method of analysis draws inspiration and guidance from (reflexive) thematic analysis (Braun and Clarke 2006, 2024). After transcription and preliminary review, 13 initial codes were generated from a close reading of the transcripts by one author (anon). Many of these codes derive from the interview schedule (Miles and Huberman 1984; Rubin and Rubin 2011), although this phase of data analysis had an inductive component as other issues emerged, such as concerns related to professional standards, and aspects of professional training. Two authors (anon and anon) independently reviewed all the transcripts and the initial codes. In reflecting on these accounts we noted interesting points that relate to studies of professional jurisdictions. To pursue these interests, we reviewed all codes to further unpack different aspects of professional work, and how the tools played into those different forms of work.

We iteratively developed the themes through a collaborative analytic process, with the aim of generating a rich description of the accounts offered by the participants. So, we produced seven categories that, in turn, and after further refinement and review, became seven sub‐themes for two overarching, inter‐related but analytically distinct, themes: *partially discarding routine work, while maintaining professional authority*; and *re‐focusing on expert work, while enhancing professional autonomy*. We used the process of developing, and subsequently summarising, these themes to reflect on the responses of HCPs to the AI tools for auto‐contouring and to explain their overwhelmingly positive reaction to these tools.

## 5 Findings

### 5.1 Professional Experiences With AI Contouring

Before discussing the two principle themes of our findings it is important to situate them within an overall context of support for the AI auto‐contouring tools by the HCPs. A question that was useful in this respect was to ask the respondents what they thought the impact would be for them if the AI tool was taken away. The responses to this question were very similar across all sites and all professional groups, with claims that the potential removal of the AI tool would have significant negative consequences. As the following interviewee stated:

We cannot do without AI, nowadays at this point. Yeah we can’t do without AI.

(Oncologist, D07)

A clinical scientist explained why there was this universal support for the AI tool:

I think I haven't encountered any oncologists who have been resistant to any of this so far, I think they're just quite happy to have anything that saves them a bit of time because they're a very busy group.

(Clinical scientist, A05)

This contrasts to the many visions and reports of AI as disruptive and transformative. The integration of these AI tools was experienced as relatively unproblematic and indeed welcome.

Furthermore, whereas managers often imagine that AI tools will increase the efficiency of health services due to its time saving properties (Scarbrough et al. 2024), this potential was not experienced in our study at the level of the patient journey. Although it might be assumed that saving time on the task of contouring would lead to reductions in the length of the patient journey, the experts we interviewed raised a series of complicating factors. For instance, they spotlighted the increased demand for services which hampers opportunities to systematically reduce the NHS cancer breach dates:

we work to breach dates [NHS cancer targets]. And so those dates remain the same with and without AI segmentation. So, in that way you could argue it’s not speeding up patients' treatments, but our workload has increased. We’re treating more patients than we were in the past. So, if we didn’t have [AI] segmentation we could only do that and not, without delaying patients more than we’re allowed to, if we hired more staff.

(Clinical scientist, D02)

Relatedly, they noted other factors that bear upon the patient journey, whether in terms of the availability of clinicians to review and sign off contours and wider plans, or in terms of the availability of limited equipment time that constrains the number of patients who can be treated in a day. So, although auto‐contouring tools might offer benefits in one part of the workflow, they are not seen to straightforwardly stimulate the wider changes necessary to realise time benefits for patient journeys. This suggests that simply implementing AI tools will not automatically result in increased efficiency, more complex service reengineering would be required.

Nevertheless, our data do not reveal resistance to the implementation of the new technology, unlike other studies of AI in healthcare (e.g., Gillner 2024; Scarbrough et al. 2024; Carboni et al. 2023; Petrakaki et al. 2012; Dupret 2017). Instead, across all five sites, the professionals we interviewed unambiguously and unanimously reported the benefits of working with AI auto‐contoured scans. This shared perspective provides an important context for the remainder of our findings which explore the reasons for these positive views across the professional groups.

### 5.2 Partially Discarding Routine Work While Maintaining Professional Authority

In comparison with manually contouring organs‐at‐risk from scratch, professionals highlighted the task time savings when using the AI tools. They also consistently referred to the routine, or even ‘tedious,’ character of the task of manual contouring:

Before AI this process of manually drawing round organs on a CT scan was done entirely by humans and it’s a long and tedious and boring process and it would take between half an hour and two hours per patient. The AI tools can do this in five or ten minutes without any direct supervision, so once they’ve done it … we’ll review it, but that’s basically just removed a 30–120 minutes task that quite highly trained, quite in demand people had to do.

(Clinical scientist, A01)

These kinds of positive account of the impact on the task of contouring were consistent across all professional groups and sites. Their broad estimates suggest considerable reductions in time devoted to contouring, whether in relation to the large time savings in contouring a specific complex structure, or smaller time savings accumulated across multiple instances of contouring simple structures. The interviewee stresses that this time saved is of particular value, because the people doing the task are ‘quite highly trained’ and ‘quite in demand.’ Importantly, then, although some respondents stated that they enjoyed doing a certain amount of manual contouring because they found it quite relaxing, most felt it was a part of their role that they were happy to discard, as it was characterised as routine and tedious work:

for me, it takes a huge pressure off, I mean, it has a time pressure, but it’s also tedium, pressure of outlining the spinal cord.

(Oncologist, C08)

Nevertheless, the interviewees were not uncritical about the limits to the tools. They reported recurrent occasions when the tool did not adequately appreciate aspects of context resulting in basic errors, which resonates with Lebovitz et al.’s (2021) differentiation of AI's ability to *know‐what* but its inability to *know‐how*. To illustrate, the following oncologist refers to the AI tool as ‘the thing’:

so about 75% you don’t really need to do anything … probably 25% of it you need to tweak and probably about 2% of them you need to really start over again and just completely ignore what **the thing** has said because it just completely goes off piste.

(Oncologist, E06)

To elaborate on what going ‘off piste’ means, many of our interviewees identified specific problems that would arise by drawing on their everyday experience of OAR contouring. Often these examples would refer to challenges in contouring specific structures—for instance, organs in the pelvic region. Also, they reported that post‐operative patients present a challenge to AI tools as their anatomy might be rather unique—so, ‘post op, things get in a muddle’ (Oncologist, C08). Issues also arose in relation to aspects of context that were not readily available to the AI tools, such as differences between normative expectations and local practice:

here we put some patients prone because one of our doctors like prone and [the AI tool] does some miraculously weird things like put the femoral head outside the body on a little square… Also it doesn’t get your left and right once you put them prone, it swaps everything.

(Radiographer, D03)

We would argue that these findings have similarities with some aspects of Timmermans' (2005) work on experiences of introducing practice guidelines into different domains of healthcare. Importantly, he noted that the impact of practice guidelines on clinical behaviour was not uniform, but was dependent on various factors such as their quality, the particular characteristics of the HCP and the clinical context. Similarly, here, we find that the specificities of local practice and procedure has relevance for the types of problems or errors that emerge.

In various ways, HCPs see the technology as inferior to human expertise, with AI being seen to make basic errors, which provide grounds for them to argue both (i) the tool can only be delegated low status work and (ii) that professionals are required to maintain oversight to review and, if necessary, apply manual corrections, thus maintaining the HCPs' ultimate authority over this work. There is some danger of a professional simply accepting the AI‐produced contours (or AI output more broadly, see Anthony 2021), but this seems to be mitigated by the senior professionals' commitment to maintaining professional training in manual contouring for those entering the profession. Our interviewees, therefore, suggested that continued professional training in manual contouring is critical to ensure the enduring quality of oversight, review and jurisdiction of AI‐generated contours:

it hasn’t done away with people, you know like the human side of it totally, we've still got to train them [junior and student radiographers] and get them trained in defining something manually themselves first before they go on to using [AI tool] and accepting what it’s giving us basically. You can't just accept, you’ve got to have people who are competent to plan and outline stuff and recognise when something’s not right that needs amending.

(Radiographer, B03)

In spite of treating the system as somewhat inept, HCPs emphasised the benefits of receiving a draft set of contours rather than always starting from scratch, treating the AI as a subordinate assistant that could do the ‘dirty work’ (Hughes 1951) of preliminary contouring. In addition, our interviewees spoke about the way the tool added few, if any, new steps to the standard workflow, ‘it just magically appears on my list’ (*Oncologist, D07*). Even learning to use the system was, ‘fairly quick because it is just teaching people how to send the data to the cloud and pull it back in’ (Radiographer, C01). Therefore, little additional work on the part of the professional was needed to learn how to use the tool and accommodate it in their established practice and authority. As only the ‘tedious’ aspects of the task are seen to be discarded, the threat to epistemic authority often found in studies of AI and the professions (e.g., Scarbrough et al. 2024) is side‐stepped.

Also, the output is presented in a very familiar way; the contours are either appropriate or not with regard to local and professional standards, so there is seemingly no ‘black box’ hiding an account for the contours generated. This means that the interpretation of the AI output is more straightforward. As the tool's output is not opaque, there is less danger of the uncertainty found in other studies (Lebovitz et al. 2022; Hartswood et al. 2003). Our professionals did not need to devote time to trust the output, as they viewed themselves as accountable for the accuracy of the contours (oncologists) or deferred accountability and authority to oncologists reviewing contours at a later stage (radiotherapists, dosimetrists and clinical scientists). Indeed, they recurrently noted that the AI tool made the errors of the inept, providing a case for continued human oversight and professional jurisdiction (Abbott 1988).

In summary, there are several critical issues here. First, professionals unambiguously and unanimously reported benefits of the contouring tasks that the AI tool delivered. Second, the task of contouring is broadly conceived as tedious or routine by the HCPs and therefore open to potential discard (Kessler et al. 2015). Indeed, HCPs reported notable benefits in discarding the unpleasant (Hughes 1951) preliminary phase of contouring to the AI tool. Third, they reported limited additional transaction costs, as the AI tools are embedded within existing workflows without the need for extra steps or burdensome training. In fact, clinicians reported that they reviewed contours in exactly the same way whether the preliminary version had been produced by AI or a colleague. Nevertheless, they saw the continued relevance for training and professional development in manual contouring, to ensure that core professional skills are not lost and their jurisdictional authority was maintained (Abbott 1988). Finally, the AI is treated as rather inept and dumb, in that it is seen to make very basic errors, some of which fail to recognise simple contexts of data production or local practice. This means that the professionals firmly position themselves as the experts in this human–computer interaction, delegating low status work to the tool (Kessler et al. 2015), and position the tool to *assist* rather than *replace* them. Furthermore, offloading some of the drudge and unpleasant work to the AI tool enabled them to complete this tedious task more quickly and efficiently in most cases, while ensuring that they retained jurisdictional authority over contouring work (Hughes 1951; Abbott 1988). The contours continued to be ‘signed off’ by the oncologist, who maintained overall professional control of, and accountability for, the contours, so there was seen to be, in Abbott's (1988) terms, no jurisdictional contest.

### 5.3 Re‐Focusing on Expert Work While Enhancing Professional Autonomy

(Darzi 2024, 103) suggests that ‘The NHS, in common with most health systems, continues to struggle to fully realise the benefits of information technology. It always seems to add to the workload of clinicians rather than releasing more time to care.’ However, our case offers a contrast. Some respondents invoked the post‐pandemic context in which the number of oncology patients has steeply risen due to backlogs, while staff shortages are common. As a result, many of our interviewees argued that the introduction of these AI tools have been vital in meeting this extra workload and enabling them to process the patients in a resource constrained environment.

A small number noted improvements to their work‐life balance. However, HCPs often work in a ‘time famine’ (Perlow 1999), experiencing the feeling of having too much to do and not enough time to do it. Maybe as a result, interviewees rarely reported lower workloads, but rather reported better quality experiences in the nature of their work. Indeed, the interviewees reported a series of opportunities offered by the use of these AI tools that consistently related to their professional status and identity, including their professional project to improve the quality of patient care.

These tools were seen to offer opportunities to focus on more specialist aspects of their expertise, which drive forward their professions. These accounts stand in contrast to often cited fears of the deskilling of work by AI. One interviewee crystalised the opportunity to re‐focus on other aspects of their expertise rather than the seemingly more low level skills delegated to AI, as follows:

And people worry about it replacing clinicians—I don’t think our expertise is drawing around lumps and bumps!

(Oncologist, E06)

Many respondents discussed other aspects of their work that they now secured greater autonomy over and were able to assign greater priority. For example:

I can actually just … focus on other things: trying to streamline the service, try and improve the service which I never actually got time for before. I was just kind of doing my day job. Whereas actually I kind of want to be pushing things forward. And it just gives me that extra time then to be able to focus on those extra bits that I didn’t get time to before.

(Radiographer, C04)

Thus, they focus on aspects of their work that they see as higher status and closely aligned to more expert areas of their professional projects (e.g., service improvement, research etc.).

Furthermore, HCPs revealed that the AI tools have enabled a more significant re‐focusing on care for individual patients. For instance, they reported that individual patients receive *more precise* forms of treatment following the introduction of the AI auto‐contouring tools, whether in terms of greater emphasis on treatment planning or, indeed, contouring the tumour, which remains the sole preserve and authority of the oncologist:

designing the plan is integral and that often takes a lot of time and we often find that we don’t have a lot of time to do that, so if we can free up half an hour from the voluming that’s another half an hour that we can spend in perfecting the plan, so it gives you, certainly for the patient it would be a better clinical outcome.

(Radiographer, B01)

This emphasis on more expert tasks, allows the HCPs to introduce new and better treatments that further enhance the status of their profession. In one of our sites, it was noted that the introduction and use of adaptive radiotherapy has been partly facilitated by the speed with which AI tools can re‐contour the OAR. In adaptive radiotherapy, a new plan is produced for the patient each day to ensure that the plan more precisely limits the doses of radiation to the OAR. In turn, this aims to reduce long term side effects and improve quality of life. Aside from the fact that fewer patients could be treated using adaptive radiotherapy without the AI tools (as each individual treatment slot would need to be extended), adaptive treatment requires fairly fast re‐planning to be most effective. As one radiographer explained:

there’s a time pressure when I’m contouring them that I have to save time. So AI obviously can be a godsend in a little way because obviously it’s saving my time of that patient, that I know if their bladder’s going to fill, even if I’m an extra five minutes, that bladder filling can be significant and push the area that we want to treat out of where I’ve actually told the computer where it is today. So yes it’s a huge impact, even a few minutes of saving time.

(Radiographer, D05)

Similarly, interviewees reported that opportunities for more precise auto‐contouring have led to other advanced techniques. One site reported the use of ‘deep inspiration breath hold’ treatment for breast cancer, in which the patient breathes in deeply to separate the breast from the lung and heart, thereby lowering the radiation dose to the heart and lung. One clinical scientist suggested that, ‘using [AI tool] and all the improvements it's made, we can now offer that to many many more patients’ (B04).

This analytic theme illustrates how the introduction of the AI tools has had a profound, and perhaps surprizing, impact on professional work. Following the introduction of AI tools, professionals reported that they were able to re‐focus their efforts to ensure greater emphasis on activities that enhanced their professions and professional projects. They welcomed this enhanced autonomy. This drive to outsource tedious, or ‘dangerously routine’ (Abbott 1988; Hughes 1951; Bosk 1979; Kessler et al. 2015), aspects of contouring are in line with our broader understandings from the sociology of the professions. Moreover, it enhanced HCPs' professional autonomy to embrace tasks they conceived as more expert and higher status (cf. Kirkpatrick et al. 2011). Interestingly, the HCPs stated that these higher status tasks focused on individual patient care, but not in ways that might commonly be anticipated, such as face time with patients. Rather they highlighted greater opportunities for an enhanced quality of personalised treatment planning. They also reported that the development of, and access to, certain forms of advanced care for individual patients had improved as a result of the introduction of the AI tools.

## 6 Conclusion

Rather than the many visions and reports of AI as disruptive and transformative, the integration of AI tools for OAR contouring has been experienced as relatively unproblematic by the HCPs we interviewed. AI auto‐contouring tools are overwhelmingly popular among radiotherapy HCPs and, unlike other studies of AI in healthcare (e.g., Gillner 2024; Scarbrough et al. 2024; Carboni et al. 2023; Petrakaki et al. 2012; Dupret 2017), our data did not reveal significant professional resistance. Overall, this resonates with scholars (Pakarinen and Huising 2025) who rebut the idea that professions are *bound to resist* AI in all cases. They rightly note that the professions depend on technologies to do their work and will often work to create or improve the quality of such technologies.

Our findings offer two key contributions to sociological studies and debates. First, as AI increasingly intrudes on the white‐collar workforce, our findings provide a striking reminder of enduring relevance of key insights from the sociology of the professions. Indeed, this body of work provides distinctive resources for us to reflect on the ways in which AI is embedded (or not) within the system of professions. We have noted that the extant literature clearly articulates the work of the professions to delegate routine, undesirable, or tedious tasks to less expert staff (Abbott 1988; Hughes 1951; Bosk 1979; Kirkpatrick et al. 2011). (Kessler et al. 2015, 748) frame this as a ‘specialist‐discard logic,’ where HCPs are relieved ‘of the ‘burdensome’ or ‘dangerous’ routine, allowing them to concentrate on more technically advanced tasks and, in so doing, advancing their professional project.’ This is clear in relation to OAR contouring and it resonates with the optimistic vision of an AI future, described by Anthony et al. (2023), that argues that humans will collaborate effectively with AI (Wilson and Daugherty 2018) when it takes on routine tasks, allowing professionals to take on ‘higher order’ work (Susskind and Susskind 2015). In our case, HCPs regained autonomy to re‐focus their work on higher status (research and service improvement) and higher value (precision treatment and tumour contouring) activities.

However, the specialist‐discard logic is not fully deployed in our case and this presents a novel twist on the concept of discard. Whereas entire tasks are often seen to be delegated in prior studies and literature, we find a *partial* discard of the contouring task in our findings. Although the first draft of the task is delegated to the AI tool, the task still remains with, and returns to, the HCPs involved for oversight and, where necessary manual correction. This partial discard offers opportunities for HCPs to retain authority and jurisdictional control, enhance their broader professional autonomy, and yet outsource the tedious first steps of the task. If the AI output is good enough, it is simply approved, otherwise the HCP can correct or refine. Indeed, OAR contouring has witnessed earlier versions of this partial discard, as radiographers previously took on first drafts on contouring a select number of organ structures from oncologists. Nevertheless, the oncologist retained jurisdictional control, whereas outsourcing preliminary drawings. This suggests that partial discard is vitally important to boundary work, and almost certainly extends beyond the study of technology to the analysis of competition between occupational groups. If we reflect on Noordegraaf's (2020) interest in ‘the question of how professional action can be related to others and outsiders and remain ‘knowledgeable’, ‘autonomous’ and ‘authoritative’ at the same time,’ the partial discard offers one way in which professionals can manage the existential threat of AI (or technological disruption more generally). The AI tool is used to support the HCP, but does not challenge their authority or jurisdiction, indeed it is not even required to undertake the task. The professionals retain overarching control over the task, which may partly explain its popularity.

This takes us to the second contribution, which is to offer a corrective to numerous lay and sociological accounts of ‘artificial intelligence’ as a homogeneous technology. There is a wide array of different types of AI being used in healthcare and so it is not particularly helpful to consider how HCPs might react to the introduction of AI as an undifferentiated whole. The processes of ‘domestication’ (Johannessen et al. 2024)—which refers to the ‘taming’ of new technologies—are similarly not homogeneous. As with other studies of healthcare technologies (Pols and Willems 2011), the processes of domestication will involve the tool being fitted to the local practices of the users. The added complexity here is that not all AI tools are equivalent.

Although some discriminative AI tools are resisted as they are seen to challenge professional diagnostic reasoning and professional jurisdictions (Abbott 1988), the OAR tools in our case are positioned in the workflow as subservient and do not interfere with professional authority (Huising 2023) because the oncologist remains dominant. The professional casts the AI tool as a dumb assistant that cannot be trusted to work in an autonomous way but instead needs to be directed and authorised by the professional. This offers an interesting possibility—this very ‘clever’ AI system is, at least in part, experienced so positively, because it is seen as ‘dumb’ and non‐challenging and, thus, there are no jurisdictional contests (Abbott 1988). In addition, the concerns with the ‘opacity’ of AI output that is found in prior studies (e.g., Lebovitz et al. 2022; Hartswood et al. 2003) is not relevant here, as the output is *descriptive* rather than *diagnostic*—the validity of the contours can be assessed at face value. Taken together, these points suggest that it is not AI per se that professionals may find threatening but it is the specificities of the AI in the contexts of local professional practice that is critical. Indeed, the acceptance of the tool seems, in part, because it embodies a model of AI as a ‘system for experts’ (Whalen 1995), rather than an ‘expert system’ designed to challenge, or even replace, professional vision.

More generally, then, we would suggest that in order to pursue sociological studies of AI, we need to move beyond blanket categories of AI to examine instead the specific technology that is being implemented, the organisational contexts into which it is being introduced and the professional contexts in which it is embedded. This has relevance that goes well beyond healthcare to studies of how technologies are ‘made at home’ in work as it is practiced (Sacks 1992). The approach can offer insights into the technologies that professions may be most likely to welcome, where it is likely to be those that are positioned (by designers, managers and professionals) as tools to augment and support, rather than challenge, compete or supplant their conceptions of expertise and authority.

## Author Contributions

**Juan I. Baeza:** conceptualization, data curation, formal analysis, investigation, methodology, project administration, validation, writing – original draft, writing – review and editing. **Jon Hindmarsh:** conceptualization, funding acquisition, formal analysis, investigation, methodology, validation, writing – original draft, writing – review and editing. **Angie A. Kehagia:** conceptualization, funding acquisition, writing – review and editing. **Anna Barnes:** conceptualization, funding acquisition, writing – review and editing.

## Funding

This study was part of Health economic evaluation of AI‐enabled auto‐contouring tools for radiotherapy treatment planning: HE‐AI‐RT) funded by the Artificial Intelligence in Health and Care Award, one of the NHS AI Lab programmes, to King's Technology Evaluation Centre (KiTEC) at King's College London. The competitive award scheme is run by the Department of Health and Social Care. The AI Award has made funding available to accelerate the testing and evaluation of artificial intelligence technologies which meet the aims set out in the NHS Long Term Plan.

## Data Availability

Research data are not shared.
